# Guidance on stakeholder engagement practices to inform the development of area-wide vector control methods

**DOI:** 10.1371/journal.pntd.0007286

**Published:** 2019-04-25

**Authors:** Delphine Thizy, Claudia Emerson, Johanna Gibbs, Sarah Hartley, Lydia Kapiriri, James Lavery, Jeantine Lunshof, Janine Ramsey, Julie Shapiro, Jerome Amir Singh, Lea Pare Toe, Isabelle Coche, Benjamin Robinson

**Affiliations:** 1 Department of Life Sciences, Imperial College London, London, United Kingdom; 2 Institute on Ethics and Policy for Innovation, Faculty of Humanities, McMaster University, Hamilton, Ontario, Canada; 3 Keystone Policy Center, Keystone, Colorado, United States of America; 4 Department of Science, Innovation, Technology, and Entrepreneurship, University of Exeter, Exeter, Devon, United Kingdom; 5 Department of Health, Ageing and Society, McMaster University, Hamilton, Ontario, Canada; 6 Hubert Department of Global Health, Rollins School of Public Health, Center for Ethics, Emory University, Atlanta, Georgia, United States of America; 7 MIT Media Lab, Massachusetts Institute of Technology, Cambridge, Massachusetts, United States of America; 8 Department of Genetics, Harvard Medical School, Boston, Massachusetts, United States of America; 9 University Medical Center Groningen, University of Groningen, Groningen, the Netherlands; 10 National Institute of Public Health, Cuernavaca, Morelos, Mexico; 11 Center for the AIDS Programme of Research in South Africa, University of KwaZulu-Natal, Durban, KwaZulu-Natal, South Africa; 12 Dalla Lana School of Public Health, University of Toronto, Toronto, Ontario, Canada; 13 Research Institute of Health Sciences, Ouagadougou, Burkina Faso; 14 Emerging Ag, Calgary, Alberta, Canada; Makerere University, UGANDA

## Introduction and purpose

These recommendations on practices for stakeholder engagement build on the knowledge and experience of practitioners and subject-matter experts from a large variety of fields. They aim to provide teams involved in the development of area-wide vector control methods with guidance on how to design community and stakeholder engagement programmes as part of their development pathway.

Area-wide vector control methods are not new in concept [1]. For example, the use of natural predators for biocontrol of agricultural pests or public intervention for the treatment of water bodies with larvicides are current area-wide applications. Thus, new approaches under development—such as those using sterile-male techniques, *Wolbachia*, or gene drive—can build upon well-established development pathways.

These methods offer the benefit of providing vector control for all inhabitants of a specific treated area without individual or group biases related to economic means, level of education, etc. Although this benefit can be a great advantage, multiperson or community-applied interventions may not offer individuals the chance to ‘opt out’ of a home or area receiving the intervention [2]. There may be aspects of the research in which individuals can choose to participate or not (for example, during the collection of mosquitoes or other insects from houses), but the research or technique developed will, at some stage, require the deployment of the tools in selected sites, and residents of those sites may not be able to ‘opt out’ of these phases in the same way that individuals can decline to be part of a vaccine field trial. Therefore, although these methods may differ greatly in scope and impact, the processes for their development and use share commonalities that provide a basis for asserting a broadly applicable framework for engagement. The framework set out below attempts to leverage these commonalities to provide a starting point that may be useful to a very wide variety of projects working in this field while retaining sufficient flexibility to be tailored as appropriate to local circumstances

Some methods, such as gene drives and environmentally persistent *Wolbachia* methods, involve modifications to vector species that could persist and disseminate through a population over time and space. This means that the scale of the human communities affected can be quite large fairly early on in the development process and may persist or increase over time.

Thus, testing of area-wide vector control methods does not conform to the familiar clinical trial model employing individual informed consent [3], and a different set of research ethics considerations is required. Trial authorisation by the hosting community and, potentially, peripheral communities that may be affected may be necessary. Therefore, identification of key stakeholders and community representatives is vital, and early and ongoing engagement with them is essential. This type of engagement can enable the sharing of knowledge, experience, and perceptions between the project and its stakeholders to help ensure that the pathways for development respond to the expectations of communities and other stakeholders, that the tools developed are ultimately effective and appropriate and meet the needs and preferences of users and other potential beneficiaries, and that accountability is established for trial outcomes and eventual disease mitigation.

The recommendations may serve not only for projects involved in the development of new area-wide vector control methods but also as a benchmark to shape the expectations of a wider range of stakeholders, including project funders, communities, the media, and policy makers and regulators, about what projects will and should deliver. More broadly, shared recommendations can help facilitate the alignment of approaches of different projects and provide a clear pathway for other projects entering the field. These recommendations also describe ways in which projects can engage with stakeholders to meet other requirements for their research, including community acceptance for specific activities or phases of the research that may be necessary for the research to be able to proceed.

However, the ability to implement these recommendations hinges on the availability of appropriate funding. Many of the suggested activities and approaches will be resource intensive. Therefore, funders are encouraged to make adequate supplies available for stakeholder engagement at every stage of the development process.

## Context for the development of these recommendations

Recent technological developments in the field of vector control have led to significant interest in the importance and practice of stakeholder engagement for area-wide vector control interventions. The 2016 report *Gene Drives on the Horizon*: *Advancing Science*, *Navigating Uncertainty*, *and Aligning Research with Public Values*, published by the National Academies of Sciences, Engineering, and Medicine, includes an explicit framework and recommendations related to stakeholder engagement in the deployment of gene drive vector control techniques [4]. These discussions have drawn renewed attention to questions surrounding stakeholder engagement activities for area-wide interventions and have dovetailed with concerns surrounding the safety and acceptability of gene modification technologies.

It is important to note that an existing body of knowledge on practices for stakeholder engagement has been developed across many sectors over the years, including in the extractive industries [5–6], conservation and the environment [7–9], and public health [10–15]. Elements of this body of knowledge are applicable to engagement for area-wide vector control methods.

There are thus existing foundations on which to build engagement programmes for area-wide vector control. However, changing public attitudes towards policy makers, science, and information and growing expectations of public engagement in decision-making have contributed to changing the environment in which engagement now takes place [16].

## Method

These recommendations were developed over the course of a year by a multidisciplinary team of experts and practitioners with funding from Target Malaria and the Foundation for the National Institutes of Health. The process began with two 4-week online consultations wherein participants selected on the basis of relevant experience (in this case, meaning practical experience in designing, implementing, or informing stakeholder engagement processes for projects seeking to deploy area-wide vector control interventions or the pursuit of an academic career related to the same) were asked to submit and comment on personal experiences, case studies, and published materials relevant to stakeholder engagement for area-wide vector control methods. Submissions were presented in writing in response to specific prompts and were composed of reflections synthesising key lessons from past experience and research related to stakeholder engagement as well as published materials that provided further detail or were particularly illustrative of specific points. Individual submissions were shared with all participants, and all participants were invited to provide comments on any previous submission of commentary. All participants are included either among the paper’s authors or among the contributors listed in the Acknowledgments section.

These submissions were then summarised and used as the basis for an in-person, 3-day workshop, during which an initial version of the guidance document was produced through an interactive and highly collaborative drafting process. The workshop was held from November 15 to 17 in Reston, Virginia, United States of America, bringing together 23 experts, all of whom are included either among the authors or contributors list of this paper. The paper was then sent out to an external group of reviewers to gather additional inputs and comments. A final draft was then submitted to all the contributors. Contributors are acknowledged at the end of the article.

Contributors drew on their practical experience as well as their knowledge of the relevant literature to identify commonalities in successful stakeholder engagement approaches and derive broadly applicable recommendations useful in many different contexts and at many different scales to area-wide vector control.

These recommendations represent an attempt to address many of the issues discussed in the report *Gene Drives on the Horizon*: *Advancing Science*, *Navigating Uncertainty*, *and Aligning Research with Public Values*, published by the National Academies of Sciences, Engineering, and Medicine. As a result, the framing and language used will in many cases be in line with formulations found in that publication.

## Definitions

- Communities: Groups of people who live within the geographical location or biologically relevant proximity (e.g., flight distance of a targeted insect vector) to a potential site where research is taking place or where field releases may take place such that they have tangible and immediate interests in the research project. Communities are included within the broader category of ‘stakeholders’. (Based on the definition used in [3].)
- Stakeholders: Organisations, groups, or persons with professional or personal interests sufficient to justify engagement but who may or may not have geographic proximity to potential intervention sites for the research project. (Based on the definition used in [3].)
- Publics: Groups who lack the direct connection to a project that stakeholders and communities have but nonetheless have interests, concerns, hopes, fears, and values that can contribute and influence decision-making about the research and possible use of the vector control intervention. (Based on the definition used in [3].)
- Codevelopment: A collaborative process of jointly designing a research pathway and its resultant intervention to reach a common goal.

## General considerations

### Funding

There are elements of project implementation that can improve the viability and success of stakeholder engagement strategies from the outset and that should be considered in the early stages of project design. One particularly important component that affects teams’ ability to develop successful stakeholder engagement strategies and to carry those out is the availability of funding and support, both from their funders and within their own teams. In the early stages of the project, a specific portion of the project’s budget should be allocated to engagement work, which will evolve with time as the project moves forward and may differ depending on the context of engagement. Projects should ensure that funding sources are fully transparent to both project members and relevant external stakeholders.

### Engaging throughout a project’s life cycle

Engagement should begin at the onset of a project, not only once a tool or method has been developed. Engagement can contribute to shaping the research and the resultant vector control methods, and it should help inform the development pathway as well as other components of the development process, such as risk assessments. The growing interest from many parties (in particular, health authorities and regulators) in having projects codevelop interventions with stakeholders requires engaging in a dialogue with stakeholders at an early stage so that their concerns and expectations can help shape future project activities and outcomes. Engagement activities should continue throughout the lifetime of a project on an ongoing and incremental basis. The areas and foci of work are likely to be substantially different at different stages along the project timeline, and stakeholders should be granted the opportunity to reflect on these changes.

Projects should therefore ensure that stakeholders’ role with regard to research and/or technology development is clear to all involved, particularly when adopting an approach focused on codevelopment or complementarity (in which stakeholders may be involved in decision-making processes). Ensuring that the terms, limitations, and expectations of stakeholder participation are understood helps to avoid distrust and the withholding of information while promoting honesty and transparency. Clarity can also help address issues of power dynamics between teams and stakeholders.

As far as possible, projects should provide for the possibility of stakeholder input on the means and expected outcomes at each stage of the engagement activities. Finally, they should recognise that interests, stakeholders (including communities), etc., will evolve during the research/development process, and therefore the process of stakeholder analysis and involvement in the engagement process should be dynamic.

### Project team member involvement

The composition of the team developing the engagement methods needs to reflect the diversity of activities that will need to be undertaken and may vary according to the stage of development. An interdisciplinary team should include a dedicated engagement group with appropriate skills. The particular set of skills deemed appropriate not only will depend on the specific investigational product, context, organisation, and objectives of the team but may also include mediation, facilitation, multiparty consensus building, social work, community mobilisation, communications, youth outreach, conflict resolution, public relations, etc. Teams should include experts on a diverse range of topics, including local culture, economics, politics, and history. Experts are needed to collaborate and build capacity among the teams across project locations.

Such a team should also ensure that scientists and researchers have a clear and explicit role in engagement activities and a clear understanding of the rationale behind them. They should provide stakeholder engagement experts with support related to the technical and scientific aspects of the project and be actively involved in discussions with stakeholders in order to address technical questions and concerns and ensure direct two-way communication between project teams and stakeholders. This type of engagement requires providing support to the scientists and researchers to ensure that their intervention is aligned with the values and approach of engagement.

### Mutual respect

Engagement activities will only be successful if they are perceived by all parties as worthwhile exercises undertaken in good faith. The tone of these activities will therefore be a key determinant of positive outcomes. Projects should therefore allow for sufficient notice and time for a meaningful engagement encounter between project teams and stakeholders in user-friendly and accessible venues that break down expert/layperson power dynamics. In addition, they should establish and sustain an institutional attitude and culture committed to respectful engagement. Finally, they should recognise that engagement involves the reciprocal exchange of knowledge between stakeholders and the project team, acknowledging that stakeholders’ and project teams’ views are based not only on science but also on interests and values.

### Flexibility and compromise

The views of stakeholders and teams on the goals and processes of projects may not be perfectly aligned. Therefore, an effective codevelopment approach will require dialogue and compromise among the actors involved. Project teams must recognise and respect that robust involvement of stakeholders in decision-making processes may entail the cessation of some or all of the project’s activities and/or require teams to undertake research that they had not originally planned.

### Risks associated with engagement

Stakeholder engagement activities often entail risks to stakeholders and project members. Project teams should give appropriate consideration to the means of identifying and mitigating them. Relevant risks include raising unrealistic expectations among stakeholders that may undermine the objectives of the project and/or relationships between stakeholders and project members, stigmatising particular community members (e.g. those who do or, conversely, those who don’t take part in certain engagement activities), and privileging certain community members over others in the course of engagement activities, which may lead to distrust, gatekeeping, and abuse of influence.

## Responsibilities and accountabilities for research teams and stakeholders

The following recommendations are intended to assist project teams to remain responsive to stakeholder concerns, be proactive about sharing research results, and put in place robust and accessible mechanisms for responsiveness. It is important to recognise that there is often an asymmetry of accountability between project teams and stakeholders; therefore, although all those engaged should feel as if they are part of the process, each participant’s level of expectations should be made clear. Projects should also work within the existing accountability mechanisms, such as national and international legal frameworks, research ethics protocols, and human rights principles.

Where they exist, projects should seek the approval of relevant institutional ethics committees and/or regional/national committees prior to engagement in order to ensure accountability. They may also wish to consider establishing an independent ethics advisory group to provide additional advice to the project. In addition, project teams should take into account recommendations from other pertinent actors, such as regulators, public health agencies, local authorities, and religious authorities, even if such recommendations are not legally binding.

Project teams should establish clear and accessible grievance and complaint mechanisms and inform stakeholders of how they function. Stakeholders should be consulted and participate in designing the grievance and complaint mechanisms to ensure that they meet their needs.

Project teams have a responsibility to ensure that the mandates, including expectations and roles, of those taking part in research and engagement processes are clear to all parties. In order to do this, they should set expectations up front for how progress will be shared and how teams will remain responsive to concerns. They should also keep in mind that responsibility should be reciprocal. Projects should ensure that stakeholders are aware of their responsibilities as partners in a project. Developing a shared understanding of responsibilities can contribute to codevelopment of the intervention. Finally, teams should provide feedback to stakeholders about how engagement input was used and how it shaped the project in order to ensure that the engagement process is transparent to all.

## Preparations for engagement

### Preliminary considerations

Before beginning engagement (or a new phase of engagement), project teams should consider whether they have the necessary information, resources, and infrastructure in place to ensure the success of stakeholder engagement processes. A formalised engagement strategy should be created that considers the objectives and the context for engagement to select the appropriate personnel, engagement tools, formats, mechanisms, timeline, and approaches. Project teams should be sure they understand the relevant context and scope (social, economic, cultural, geographic, political) that will shape engagement. This will often involve collecting formative social science data. This can include information relating to demographics, level of knowledge among stakeholders of the issues the teams are dealing with, level of trust, preliminary attitudes, social organisation, interest and perspectives, power dynamics among stakeholders and in the project or the host institution, etc.

Project teams and stakeholders should discuss and decide together on the mechanisms for engagement and review, amend, update, and refine them as necessary on an ongoing basis. This process requires an initial sharing of information about the work and objectives of the project to help stakeholders understand what is involved. The right balance must be found between early engagement, management of expectations, and stakeholder fatigue. The engagement path should be proportional to stakeholders’ understanding of the project, the project’s impact, and the phase of development. Related to this, project teams should have a good grasp of the project development process, including relevant project timelines, in order to ensure that the engagement process is relevant to the broader work of the project and allows sufficient time for potential changes in project development pathways. Objectives for engagement should be clear to both those conducting the engagement and the audience for engagement.

Projects are motivated to engage for different reasons. Instrumental motivations and/or legal requirements should not be the sole basis for engagement. Engagement should be conceived of as a worthwhile pursuit in and of itself beyond the need to satisfy formal requirements.

### Identifying stakeholders

A clear understanding of who is likely to be significantly affected by the activities or implications of a project is vital to designing an effective engagement strategy. In addition to who those groups or individuals may be, projects also need to understand the sociocultural contexts and relations in which stakeholders may be embedded. They should distinguish to what extent stakeholders with different levels of involvement are consulted. Communities directly affected by the project will need to provide their acceptance of project activities that will have immediate impacts on them and approve the evaluation for the research to progress, whereas other stakeholders who have an interest in the use of the intervention or knowledge that could contribute to the intervention’s development and potential use need to be engaged, but their acceptance may not need to be formal. Finally, the public at large (for instance, in nonendemic countries) needs to be engaged but may not necessarily need to accept the evaluation or use of an intervention.

Projects should also ensure that stakeholders consulted represent a diverse set of interests, knowledge, and identity groupings and that a wide range of affected constituencies are represented. This diversity grants consultations a high degree of communal legitimacy and allows them to take into account the diversity of opinions and knowledge levels that may exist among stakeholders. This is particularly important for area-wide vector control methods, as individual community members will be unable to opt out.

Moreover, engagement mechanisms should make space for a diversity of opinions, including those that may be contrary to the views of projects, without the necessity for dissenting voices to be convinced. These mechanisms should take into account intracommunal power dynamics and imbalances so that representatives of a wide range of demographic and identity groupings feel able to engage actively.

### Identifying potential partners

In many contexts, project teams are unlikely to be the sole actors intervening on pertinent issues. In addition to identifying stakeholders, a good understanding of potential partner organisations/programmes can help improve the effectiveness of engagement through leveraging synergies and building on previous efforts. Projects should therefore consider the feasibility and appropriateness of engaging with other organisations/programmes to coordinate convergence of messages or collaborations, even in early phases of the research.

## Identifying, gathering, and sharing information

Providing stakeholders with clear, balanced, and objective information with regard to all relevant aspects of the project is essential to give them a solid understanding of the intended uses of the vector control method as well as risks, opportunities, costs, benefits, alternative approaches, and relevant timelines. To achieve this, projects should ensure that teams are sufficiently informed so that they feel they have the capacity to take part actively in dialogues with stakeholders.

Knowing your audience is crucial: information and information delivery strategies should be tailored to diverse audiences and levels of understanding to ensure that they are accessible, relevant, and sensitive to as wide a variety of stakeholders as possible. This will entail providing access to information with varying levels of detail and complexity so that stakeholders are able to engage with projects at the level of depth that is most suitable for them. It will also mean taking into account stakeholder knowledge when designing and implementing engagement activities to ensure that methods and approaches are adapted to sociocultural contexts and stakeholder preferences. This is particularly important when engaging across multiple languages and cultural environments.

Project messages and engagement materials should address a wide spectrum of the attitudes and issues that exist with regard to a project or the interventions it uses. It is important to recognise that the attitudes and priorities of communities may not be aligned with those of other stakeholders or the authorities that govern them. Approaches to engagement must be tailored accordingly.

Projects should avoid omitting problematic topics or themes, including uncertainty of outcomes and risks. This will involve proactively acknowledging potentially challenging issues and navigating them with an openness to learning rather than defensiveness as well as recognising that the need for transparency in information sharing also encompasses information about lack of success or delays.

Ideally, information sessions should equip stakeholders to become effective knowledge sharers themselves, able to accurately communicate the most salient information about the project to their peers and others in their spheres of influence while ensuring that the responsibility of transmitting information does not fall too heavily on actors external to the project. Relatedly, projects should consider allowing stakeholders to physically visit project facilities or participate in some activities in order to foster knowledge of, and familiarity with, the activities of the teams. Finally, projects should ensure that stakeholders have a good grasp of the scientific development process, including relevant project timelines, and should make certain to update stakeholders and project teams with new information from both the project and the community as the situation evolves.

## Involving stakeholders

Involving stakeholders for the duration of the project helps ensure that their feedback is consistently understood and incorporated into future undertakings and that they are part of the shaping of the development pathway and its resultant intervention. Projects should therefore clarify very early on in the development process the purpose and extent of stakeholder engagement, including stakeholders’ roles, how much flexibility there is for stakeholders’ involvement, and how much teams are willing to modify research processes and activities based on stakeholder input. They should make certain that there is a clear mechanism to communicate how the project team considered stakeholder inputs and the rationale for acting upon them or not.

To this end, they should also ensure that the research process remains flexible and dynamic. This requires funding mechanisms to allow for flexibility in budget allocation to be able to be responsive to stakeholders’ feedback/input. (This could be done through contingency funding, which could be used as a budget line to adapt to evolving project needs.) If this is not possible, they should ensure that transparent measures are in place to guarantee that research will be halted should preliminary inputs indicate that the efficacy or social outcomes of the project are not meeting expectations. Part and parcel of this is being open to stakeholders’ desire to get involved in some moments but not others or on topics that might not have necessarily been in the original research plan for stakeholder involvement.

Projects should consider supporting activities that enable stakeholders to deliberate and express their collective voices. It is also often wise to consider who is best placed to respond to different questions in the project. Team members who are directly involved in engagement may not be best suited to responding to every eventuality or concern that may arise. Finally, stakeholders should be informed well in advance of the modalities and rationale for any eventual cessation of project activities as well as exit from local communities.

## Incorporating knowledge from engagement into the project’s work

Any outcomes and lessons learned relating to stakeholder engagement activities should inform modifications to current practices as well as future engagement plans to improve their effectiveness. Projects should therefore ensure stakeholder engagement mechanisms are integrated into the construction of risk-assessment models, and the outputs of risk assessment are in turn fed into future engagement activities. This should involve making provision for the independent evaluation of engagement activities and, whenever possible, making these evaluations public. Evaluation results can provide feedback and/or demonstrate value propositions to the project teams (e.g., to provide lessons learned to improve/adapt while project activities are ongoing), the stakeholders, the funders, other project partners, and/or the broader network of practitioners and researchers interested in learning about/from engagement.

Projects should ensure provision is made for evaluation at every stage of the project. The types of engagement undertaken are likely to vary according to project phase, so it is important that evaluations be undertaken at each phase so that the information collected can be used to inform future rounds of engagement. All of these activities should be accompanied by adequate documentation practices to record and preserve evaluation information regarding engagement strategies, decisions, and activities, not just for the sake of transparency but also for the sake of informing future project activities and publications. Whenever possible, consider making this information open access.

## Conclusion and next steps

Although these recommendations are not comprehensive and not all of them will be applicable in every situation the authors hope that they will be a useful tool for projects in the conceptualisation and implementation of stakeholder engagement activities in a field in which the expectations around this engagement from all parties are changing. They not only reflect and complement the conclusions of many recently published guidelines on stakeholder engagement in public health but also aim to fill a new niche by addressing a hitherto understudied aspect of the field that involves unique considerations as well as ever-greater levels of interest due to emerging technologies. In addition to providing guidance to researchers and helping to mould the expectations of other stakeholders, this set of shared recommendations can help all those working in the field orient their approaches towards a common alignment and thereby facilitate convergence towards good practices. To this end, in writing this paper, the authors wish to draw increased attention to the importance of stakeholder engagement for interventions relying on area-wide vector control strategies as well as many other public health measures.
